# DrugSynthMC: An Atom-Based Generation of Drug-like Molecules with Monte Carlo Search

**DOI:** 10.1021/acs.jcim.4c01451

**Published:** 2024-09-09

**Authors:** Milo Roucairol, Alexios Georgiou, Tristan Cazenave, Filippo Prischi, Olivier E. Pardo

**Affiliations:** † LAMSADE, Université Paris-Dauphine, Pl. du Maréchal de Lattre de Tassigny, 75016 Paris, France; ‡ Randall Centre for Cell and Molecular Biophysics, School of Basic and Medical Biosciences, King’s College London, London SE1 1UL, United Kingdom; § Division of Cancer, Department of Surgery and Cancer, Imperial College, Du Cane Road, London W12 0NN, United Kingdom

## Abstract

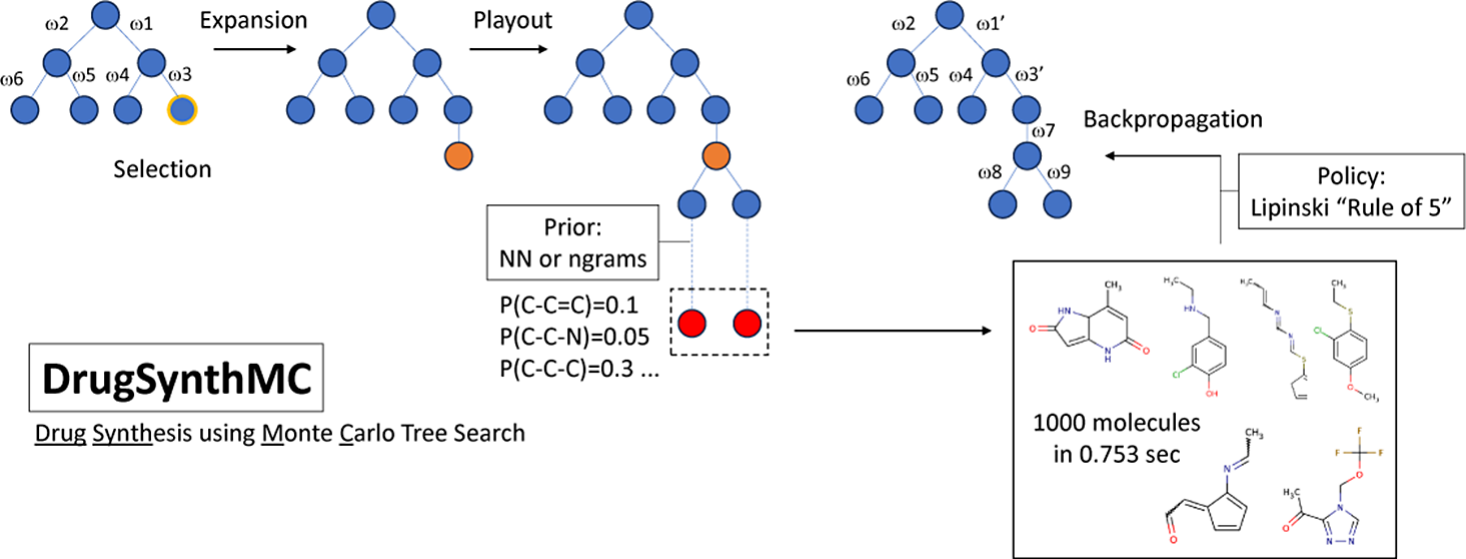

A growing number of deep learning (DL) methodologies have recently been developed to design novel compounds and expand the chemical space within virtual libraries. Most of these neural network approaches design molecules to specifically bind a target based on its structural information and/or knowledge of previously identified binders. Fewer attempts have been made to develop approaches for *de novo* design of virtual libraries, as synthesizability of generated molecules remains a challenge. In this work, we developed a new Monte Carlo Search (MCS) algorithm, DrugSynthMC (Drug Synthesis using Monte Carlo), in conjunction with DL and statistical-based priors to generate thousands of interpretable chemical structures and novel drug-like molecules per second. DrugSynthMC produces drug-like compounds using an atom-based search model that builds molecules as SMILES, character by character. Designed molecules follow Lipinski’s “rule of 5″, show a high proportion of highly water-soluble nontoxic predicted-to-be synthesizable compounds, and efficiently expand the chemical space within the libraries, without reliance on training data sets, synthesizability metrics, or enforcing during SMILES generation. Our approach can function with or without an underlying neural network and is thus easily explainable and versatile. This ease in drug-like molecule generation allows for future integration of score functions aimed at different target- or job-oriented goals. Thus, DrugSynthMC is expected to enable the functional assessment of large compound libraries covering an extensive novel chemical space, overcoming the limitations of existing drug collections. The software is available at https://github.com/RoucairolMilo/DrugSynthMC.

## Introduction

Since the 1980s, *in silico* approaches have been extensively and routinely used in drug discovery and have transformed the medicinal chemistry field,^1^ with expectation to do so even more in the future. The need for rapid response, highlighted by the emergence of resistant bacteria and, among others, the COVID-19 pandemic, has fueled the development of novel computational tools for drug design and screening.^2^*In silico* virtual-library screening (VS) is usually the first critical step in structure-based drug discovery, where the algorithm aims to predict the best matching binding mode of a ligand to a receptor.^3^ Despite the many attempts to improve accuracy of VS methods,^4,5^ the relatively limited chemical diversity of compounds in libraries reduces the ability of structure-based VS to identify hits and leads.^6,7^ Indeed, it has been estimated that only a small portion (10^6^–10^7^) of the 10^63^ drug-like molecules predicted to be synthetically accessible has been explored.^8^

Several studies have shown that screening larger libraries that expand the accessible molecules by several order of magnitude (∼10^11^) improves the rate of true high affinity (nM-pM) binders.^9−12^ To further expand the chemical space within virtual libraries, generative models based on deep learning (DL) methodologies have been used to produce molecules with desired chemical features able to bind specifically macromolecules of interest extensively reviewed in refs^13−16^.

Recurrent neural networks (RNNs) were among the first DL methods to be developed to generate SMILES, a line notation that describes the structure of a molecule.^17^ However, RNNs tend to suffer from exposure bias, and a diverse range of alternative approaches that differ in the training procedure and model architecture has been proposed. These include variational autoencoders (VAEs),^18^ generative adversarial networks (GANs),^19^ and graph-based generators.^20^ Nevertheless, even these alternative approaches have limitations and, for example, it has been reported that VAE-generated SMILES often fail to be translated into interpretable chemical structures.^21^ Furthermore, a key requirement of generative models is that designed molecules must be synthesizable. A wide range of different approaches have been used to predict synthetic feasibility of molecules, including scores based on structure complexity and similarity to evaluate synthesizability,^22^ or integrating computer-aided synthesis planning (CASP) tools as part of the design process.^23,24^ However, as recently highlighted,^25^ approaches that embed CASP tools automatically inherit CASP limitations, thus reducing chemical diversity of compounds in *de novo-*generated libraries.

In this paper, we use Monte Carlo search (MCS) algorithms in conjunction with DL and statistical-based priors to generate thousands of interpretable chemical structures and novel drug-like molecules per second. DrugSynthMC (Drug Synthesis using Monte Carlo) relies on an algorithm never previously used in chemistry/medicinal chemistry, differing from prior efforts in that it rapidly produces valid molecules, while being explainable and, importantly, requiring no training. The algorithm does not enforce or reward synthesizability (like DrugEx and SPOTLIGHT)^26,27^ or rely on synthesizability metrics during SMILES generation or selection (like SBMolGen and MolAICal),^28,29^ which has been shown to reduce diversity and novelty of generated compounds.^30^ However, the synthesizability of generated compounds was analyzed using an open-source retrosynthesis analysis tool, AiZynthFinder,^31^ and the synthetic accessibility score (SAscore) method.^32^ We show that our method generates drug-like libraries with a high proportion of predicted-to-be synthesizable compounds and efficiently expands the chemical space within the libraries. Finally, DrugSynthMC is highly flexible and could be easily tuned in the future using multiple parameters to tailor for a wide range of different chemical goals and/or create customized libraries of compounds for specific targets.

## Methods

### Search Model

The search model consists of a set of instructions defining what available moves (character addition) can be applied to unfinished SMILES. All SMILES operations start empty, and only one atom can be added at that stage. Once atoms are added, cycles and subtrees can be initiated. This search model ensures that initiated SMILES can be completed from any point of the search into a valid final SMILES, with generation being heavily restricted by the below rules. The central rule is to respect the total number of bonds that each atom can form. To do so, the number of available bonds of the last added atom in the current subtree is stored. This number is checked to compute possible legal moves and decreased when adding a character corresponding to a new atom in a different subtree level, according to the number of bonds used to connect the new atom to the previous one. To maximize the chance of generating valid molecules with drug-like properties, we scanned the FDA subset of the ZINC20 database^33^ to obtain frequency information on types of atoms and bonds involved. This information was stored in frequency matrices, allowing the search model to label as illegal moves’ bonds that were never or very rarely encountered (less than 1/10 000 of the bonds for each atom involved). It also enabled us to focus the generator on using the most commonly encountered atoms only, which are carbon (C), oxygen (O), nitrogen (N), fluorine (F), sulfur (S), and chlorine (Cl), while bromine (Br) and phosphate (P) atoms were excluded due to their relative rarity. These could, however, be easily reintroduced in the search model, along with inorganic atoms. Finally, we added shortcuts for the different bonding modes of S: bond to 2 atoms, or 4 atoms in sulfinyl, or 6 atoms in sulfonyl. Rather than learning the entire functional groups and edge possibilities through the prior, we decided to preprocess the SMILES prior to training, allocating the trifluoromethyl (W), sulfinyl (M), and sulfonyl (U) their own symbols used in building SMILES.

### Operating Principle

To give an accurate list of possible characters to append to an incomplete SMILES, the search model keeps track of several parameters concerning SMILES at any step:

(1) The depths of the nested subtrees: The subtrees are expressed in SMILES language using the ″(“symbol for opening and the ″)” symbol for closure. Termination of SMILES is not allowed unless they are back to the root tree, meaning that all open parentheses must be closed. Closing a subtree when no parentheses are open is also forbidden.

(2) Covalence bounds counts: Each subtree contains an active atom, which is the last one added at that level. It is the atom to which other atoms, cycles, and subtrees are then added. The search model keeps track of the number of available covalent bounds on the last atom of each subtree until the latter is closed by a “)”. Moves that exceed the number of covalent bonds available are not allowed.

(3) Cycle nesting: Nested cycles that share more than one bond are uncommon in drug-like molecules. Thus, only the most recent cycle is allowed to be terminated.

(4) Cycle length: More rules, not inherent to the SMILES grammar, were added to improve the drug-likeness and stability of the molecules. These included not allowing cycles smaller than 5 and larger than 7 atoms to be generated. Indeed, while these cycle sizes exist in drug-like molecules, size 4 cycles are usually hard to synthesize and unstable, while cycles longer than 7 atoms are rare.

Additionally, to avoid unnecessarily long playouts and molecules, a Boolean flag called “finish ASAP” is added. It is set to true once a certain number of characters is met and disallows certain moves, such as opening a new subtree or cycle, with some exceptions (i.e., finishing an already open subtree). This complicated set of rules is necessary to prevent the search from cornering itself due to cycles or using all of the covalent bounds available on a particular level. However, this results in a rather lengthy function enumerating the legal moves from an incomplete SMILES (about 100 lines long).

### Playouts

In Monte Carlo Search, a playout is a computationally cheap unfolding of actions from a starting search space state. Moves are selected and played until the resulting new search is terminal or no move is available. The terminal state is then evaluated and returned to be used by the algorithm, calling the playout. Playouts are a core element of most Monte Carlo Search algorithms, and the move selection process differentiates algorithms. Many algorithms use random playouts, usually when tackling NP-hard problems where expert knowledge cannot help. The prior upper confidence bounds applied to trees (PUCT)^34^ used in DeepMind’s AlphaGo^35^ employs a neural network to recommend moves to play in a game of Go. The nested rollout policy adaptation (NRPA)^34^ learns a reinforcement learning policy on the fly to select the moves. Some other applications can evaluate nonterminal states and use greedy playouts (our unpublished results). In fact, preliminary data suggest that the choice of playout mode can be more important to the success of an MCS than the algorithm used. In this study, we used the sampling method to act as a baseline to compare our algorithms. This method consists of independent playouts from the start state and ends once a molecule reaches the best possible score.

### Guided Playouts: Ngrams

Ngrams are short subsequences derived from larger sequences of characters. They were one of the first machine learning approaches, mostly used in natural language processing (NLP). Through the use of grams, it is possible to compute the statistics of every sequence of characters in a learning corpus to predict the next character in a Markovian process. Here, ngrams were generated through extracting every sequence of characters from the FDA subset of the ZINC20 database and were associated conditional probabilities used to guide the playout of an incomplete SMILES given its last characters at any step. For instance, if the learning corpus only contained “COCC” and “COCO”, the entry for “COC” would report P(C|COC) = 0.5 and P(O|COC) = 0.5. The ngrams were only used to value moves and act as a priori, following the rules of the search model. Hence, whenever the ngrams valued a move that was forbidden by the model, the move was discarded. The ngrams also used the cycle length computed from the FDA-approved compound database to guarantee the right proportion of cycles of each length in the final molecules. Storing information about the cycles’ length within the ngrams themselves would be possible but expected to be more error-prone. Therefore, we instead decided to use a probability to end a cycle at a certain length. These probabilities are 0.228 for a cycle of length 5, 0.751 for a cycle of length 6, and 0.02 for a cycle of length 7. Smaller and longer cycles were omitted for the reasons mentioned above.

### Guided Playouts: Neural Network

As previously explored,^36^ neural networks can be trained to give conditional probabilities of the next character given all of the SMILES. The neural network is then repeated on the newly extended SMILES until the SMILES is terminal, similar to recent large language models (LLMs). This method strays from the usual playouts as it is not limited by the rules of the search model, but is prone to produce invalid smiles.^36^ The same neural network can also be used as a priori, following the rules of the search model just like in the case of ngrams. This method has two advantages over approaches using ngrams: (1) taking the entire context of the SMILES into account and (2) generalization (as ngrams require an exact precursor). However, ngrams are faster, more easily explainable, and require no training.

The neural network used in the present study is the same as in ref^36^ and was trained using the same data set. It was only slightly modified to accommodate the shortcuts, with no apparent repercussions on the results obtained.

### Monte Carlo Search: Upper Confidence Bounds Applied to Trees

Monte Carlo search (MCS) encompasses a wide range of search algorithms. These differ from regular search algorithms in that they are not deterministic, but use randomness to explore search spaces too large for regular algorithms and learn guiding policies.^37^ In 2006, Kocsis and Szepesvári introduced a variant of Monte Carlo tree search (MCTS) called upper confidence bounds applied to trees (UCT) or Bandit-based Monte Carlo planning.^38^ It is now the most widely used MCTS and MCS algorithm in the literature, often in the form of prior UCT (PUCT).

UCT is a bandit-based reinforcement learning algorithm similar to Q-learning. The algorithm learns a policy and selects to go down the tree in order to balance exploitation and exploration.

Like all MCTS, UCT is composed of 4 phases:

1. Selection: Progress in the selection tree is reported according to the policy.

2. Expansion: Once a state that has not yet been explored is encountered, it is added to the tree.

3. Evaluation: A playout (or another fast algorithm) is used to evaluate the quality of the new state.

4. Backpropagation: The result of the evaluation is used to update all of the parent states visited during the selection step.

These four steps are repeated indefinitely, starting from the initial state each time, much like in Q-learning. What differs among the various MCTS algorithms is the formula of the policy.

UCT uses the following formula to evaluate child state S to select:
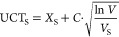
where *X*_*S*_ is the average score of state S, *C* is the exploration/exploitation constant (usually 1), *V* is the number of visits of the current state, and *V*_S_ is the number of child state S visits.

PUCT is a generalization of UCT. It uses a prior to guide not only the playouts but also the selection process, allowing us to speed up the latter with knowledge from outside this execution. PUCT uses a different selection formula:
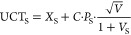
where *P*_S_ the value is given by the prior for state S.

### Nested Monte Carlo Search

Nested Monte Carlo search (NMCS) is a different type of MCS algorithm.^100^ It uses lower-level NMCS on each available move of the current state to explore the search tree and register the sequences of actions leading to the best scores. Once the lower-level NMCS returns its best routes to the higher level, it executes the next move of the best route and calls new lower-level NMCS on the resulting state. The lowest level of NMCS is (usually) a playout. Unlike UCT, the NMCS does not explore the entire search space given enough time but gains in precision as it explores the tree and is less prone to being stuck in a local maximum. This property led to generally better results from NMCS over UCT and other algorithms for optimization problems.

### Validation of the Generated Small Molecules

An *in silico* validation was performed to understand the quality and synthesizability of the generated molecules. SMILES validity, clogP, Tanimoto index, quantitative estimate of druglikeness (QED) score PAINS, and SAscores were calculated with the RDKit.^39^ The AiZynthFinder software was used to predict synthetic routes of generated SMILES.^31^ Physicochemical properties, drug-likeness, and pharmacokinetic parameters were estimated using the SwissADME and Deep-PK Web servers.^40,41^

## Results and Discussion

### Performance of Models

The aim of this work was to generate large sets of novel small molecules (i) that expand the chemical diversity of available compounds libraries, (ii) possess drug-like properties, (iii) robustly and reliably, independently from the complexity of the target, and (iv) could be used for future VS campaigns and be easily grown into larger drugs and tailored to specific targets. As such, DrugSynthMC generates, in the absence of any training, SMILES of drug-like molecules without prior targets in mind. Thus, the score function is meant to maximize only the validity and drug-likeness of the output molecules and is not goal-oriented (e.g., tailored to bind a specific biological target). The drug-likeness is obtained through maximizing the general chemical properties associated with drugs according to the “Rule of 5”.^42−44^ Indeed, easily calculated physicochemical descriptors, such as molecular weight (MW) and number of hydrogen bond donors and acceptors, have been found to correlate with the success rate of clinical trials.^45^ The function of compliance (score function) was defined as

α1 = −max(mass – 500)/500 (1) Ensures that generated compounds have a molecular weight ≤500

α2 = −max(natoms – 70)/70 (2) Ensures that the maximal number of atoms is 70

α3 = min(natoms – 20)/20 (3) Ensures that the minimal number of atoms is 20

α4 = −max(nhbd – 5)/5 (4) Ensures that the maximal number of hydrogen bond donors is 5

α5 = −max(nhba – 10)/10 (5) Ensures that the maximal number of hydrogen bond acceptors is 10

score = α1 + α2 + α3 + α4 + α5 (6) Represents the final score with maximum possible value of 5

With *mass* being the MW of the molecule, *natoms* is the number of atoms (including hydrogens), *nhbd* is the number of hydrogen bond donors, and *nhba* is the number of hydrogen bond acceptors. This formula has the advantage of being computationally cheap and requiring only a pass through the SMILES string.

To identify the most efficient method, we compared the UCT and NMCS MCS algorithms combined with different types of playouts. (i) **random**: the next character is selected uniformly randomly among the ones proposed by the search model. (ii) **enforced**: as in random, the next character is selected uniformly among the ones proposed by the search model. In order to generate compounds that are structurally valid and synthetically accessible, the score function aims to generate molecules containing the same heavy atom (C, O, N, F, S) ratios as in FDA drugs (retrieved from the FDA subset of the ZINC20 database).^33^ (iii) **ngrams**: the next character in the SMILES is selected randomly among the ones proposed by the search model, according to the conditional probabilities of the 3 character ngrams computed on the SMILES from the FDA subset. To balance the number of rings containing 5, 6, and 7 atoms, the score function uses the probability of occurrence of different rings calculated on FDA drugs in order to end a ring at a certain length. No conditional probability was used to balance the type of ring (i.e., aromatic and aliphatic, homo- and heterocycles). Additionally, characters with a probability under 0.001 are pruned, as they are judged too rare. (iv) **neural**: the next character is selected randomly among the ones proposed by the search model, according to the neural network output weight based on the incomplete SMILES input. As for ngram, characters with a probability under 0.001 are pruned. In addition, we used the sampling method as a control. This method consists of independent playouts from the start state which ends once a molecule reaches the best possible score.

For NMCS, we used a level of 3. For PUCT/UCT, we used a constant of 1. We use PUCT instead of UCT when a prior method is used (ngram and neural). Here, DrugSynthMC was run on Rust 1.59, on an Intel Core i7-11850H 2.50 GHz using a single core, to generate 1000 valid drug-like molecules in independent runs (Table S1). Generation times are not dependent on the molecule size or complexity. In all cases, the neural playout is much slower (approximately 5000 times) than that of the ngrams. The random and enforced playouts do not use a policy and show how the algorithm selection can affect the generation speed. PUCT and UCT use a timeout of 10 s, because both methods lock into local maxima induced by the shortcuts, failing to generate molecules (unless restarted immediately, turning them into Sampling). Indeed, shortcuts in PUCT and UCT increase the size of molecules and the linked scores, but often produce molecules which deviate from desirable drug-like properties.^42−44^ In contrast, UCT without shortcuts can return molecules for random and enforced generation. However, while UCT without shortcuts has generation times identical with those of NMCS and Sampling with random playouts, with enforced playouts UCT is over 100 and 6 times slower than NMCS and sampling, respectively.

The NMCS shows a clear advantage over UCT and sampling when a more complex score function is used. The design of the NMCS forces it to explore other subtrees of the search tree, thus preventing locking in local maxima. However, this feature increases generation times when using a prior method in this set of experiments. Nevertheless, as suggested by the random and enforced playout generation times, NMCS is likely to outperform UCT and sampling for specific goal-oriented generation, requiring more complex score functions.

### Validation of the Generated Drug-Like Molecules

In recent years, with the expansion of DL methods for drug design, several initiatives have been launched to assess generated compounds, which include benchmarks such as Guacamol and MOSES.^46,47^ However, these benchmarks are not suitable for methods that, like DrugSynthMC, do not exclusively rely on training data sets. Instead, we evaluated similar metrics (validity, uniqueness, novelty, diversity, physicochemical properties, and synthesizability) and used comparable tools (RDkit, ZINC databases, AiZynthFinder) to validate our algorithm.

To assess the reliability of the tool to generate valid and interpretable molecules, 10,000 generated SMILES for each of our playouts and algorithm combinations (Supplementary File 1) were translated into structure representations using RDkit.^39^ We estimated Validity as the percentage of SMILES that RDkit was able to read and correctly evaluate, and in all cases, the Validity of the inputted SMILES was 100% (Table 1), showing no syntax errors. This is significantly superior to other methods, which showed validity scores ranging from 85% for generative autoencoders to about 96% for RNN-based models (e.g., 86.24% for DeLA-Drug, 95% for ReLeaSE, 99% for MolAICal).^28,48−52^ Furthermore, DrugSynthMC shows more consistency in Validity of outputs. In fact, a recent survey of algorithms for *de novo* drug design highlighted a significant variability in the Validity of generated SMILES using different models, ranging from as low as 40.2% for E-NF (using a flow-based equivariant graph neural network (EGNN) model), to 91.9%, 94.8%, and 99% for EDM, GCDM, and JODO, respectively (all using diffusion-based EGNN models and trained on similar databases).^53^ The ability to generate novel compounds was determined by measuring the percentage of molecules in a library of 10,000 generated SMILES which was not present within ZINC-250 K^54^ (containing nearly 250,000 molecules) (Table 2), and this percentage was reported as the Novelty metric. In all cases, we see a high level of novelty within our generated SMILES. It is logical to assume that designing compounds based on general physicochemical properties of drugs instead of on a training set allows DL methods to explore a wider chemical space. Within each of the libraries generated, uniqueness was assessed by identifying the proportion of identical molecules produced within each playout and reported as the Uniqueness metric (Table 3). This shows substantial differences among the different playouts used, with ngram and neural ligands showing a higher number of replicated molecules within libraries. This is linked to the priors restricting the search space to what is more probable. To confirm this, we measured the structural similarity of compounds by determining the average edit distance.^55^ This is defined as the average minimum number of operations (insertions, deletions, and substitutions of a single character) required to transform one SMILES into another, comparing all pairwise combination of SMILES in a library of 1000 compounds. We clearly showed that both priors similarly restrict the explored chemical space (Table 4), as lower average edit distance values indicate more comparable structures, likely associated with similar properties.^56^ Conversely, generating larger drugs with a SMILES string containing ≥30 characters pushes the uniqueness above 95% with all methods. Unfortunately, it is more challenging to predict synthesizability of these larger drugs using retrosynthesis programmes.^57,58^

**Table 1:** Validity of 10,000 SMILES

	random	enforced	ngram	neural
NMCS	100%	100%	100%	100%
PUCT	–	–	100%	100%
sampling	100%	100%	100%	100%

**Table 2:** Novelty of 10,000 SMILES

	random	enforced	ngram	neural
NMCS	100.00%	99.99%	99.98%	99.96%
PUCT	–	–	99.99%	100.00%
sampling	100.00%	100.00%	100.00%	99.99%

**Table 3:** Uniqueness of 10,000 SMILES

	random	enforced	ngram	neural
NMCS	99.94%	99.17%	81.78%	87.12%
PUCT	–	–	86%	85.32%
sampling	99.83%	98%	82.58%	68.01%

**Table 4:** Average Distance for of 1,000 SMILES Compared 2 by 2

	random	enforced	ngram	neural
NMCS	17.93	14.266	12.954	13.988
PUCT	–	–	13.334	13.956
sampling	17.801	13.94	13.1	14.051

### Synthesizability of the Generated Drug-Like Molecules

**Figure 1:**
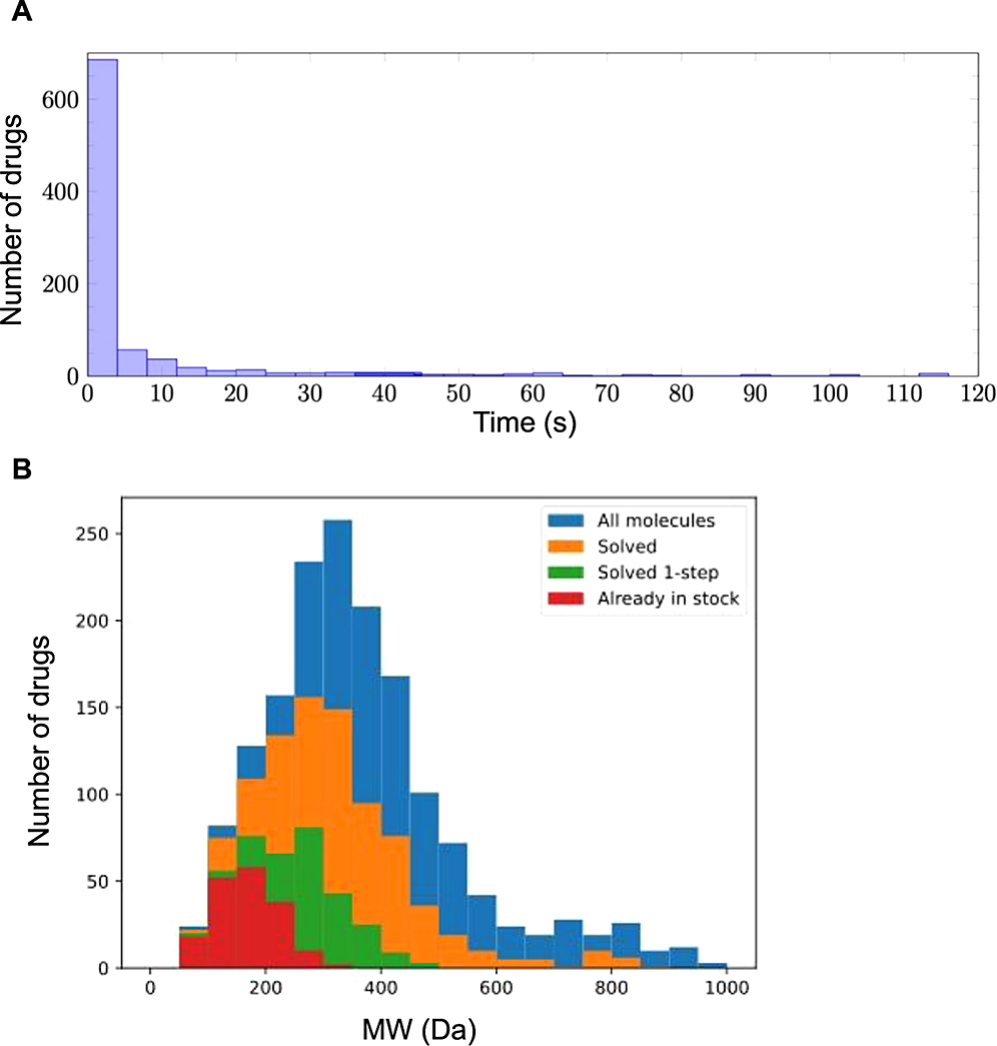
AiZynthFinder synthetic routes search: (A) histogram plot showing the number of drugs from a data set comprising 909 drugs retrieved from the FDA subset of the ZINC20 database versus AiZynthFinder synthetic routes search time in seconds. (B) Histogram plot showing the number drugs for which AiZynthFinder successfully finds synthetic routes in less than 2 min versus drugs Molecular Weight. “Solved 1-step” drugs that can be produced directly from commercially available compounds; “Already in stock” drugs that are identified as commercially available by AiZynthFinder.

A recent comparison of tools used to predict synthesizability of compounds carried out by Sanchez-Garcia et al.^59^ showed that retrosynthesis programmes tend to be more accurate than SA scores. Yet effectiveness and efficiency of different retrosynthesis tools varies quite dramatically and is still restricted in the variety of reaction types considered.^60^ Retrosynthesis programmes use a combination of search algorithms and 1-step retrosynthesis deep learning to apply reactions to a molecule and divide it into reactants available on the market. AiZynthFinder is an open-source full-fledged template-based retrosynthesis planning framework that adopts a root-parallelized MCTS,^31^ similar to ASKCOS,^61^ using PUCT. AiZynthFinder tends to successfully identify paths to synthesis in less than 2 min.^31^ Indeed, this was consistent with our retrosynthesis analysis of drugs retrieved from the FDA subset, showing that AiZynthFinder finds routes for the majority of molecules within the first 2 min of search (Figure 1A). Hence, 2 min was chosen as the maximum search time for the retrosynthesis assessment of sets of 1000 molecules generated by our algorithm. As mentioned above, there is a connection between the number of characters in a SMILES and success in identifying retrosynthesis paths. We noticed that the larger the compounds generated, the less likely AiZynthFinder successfully completed a search within 2 min or more. Indeed, AiZynthFinder finds retrosynthesis routes for ∼60% of the 1615 FDA-approved drugs and tends to fail for larger molecules (Figure 1A,B). Therefore, as proof-of-principle study, we chose to generate lower MW drug-like compounds, at the expense of uniqueness, to show the ability of our approach to generate synthesizable compounds. Due to the similarities between the algorithms when using neural or ngram playouts and, as shown above, the overall better performances of NMCS, we focused on the molecules generated by this latter method. Table S2 shows the difference in proportion of molecules predicted to be synthesisable depending on the type of NMCS playout used. The ngram playout is the one that generates the largest number of synthesizable compounds. The predicted 32.2% synthesizability is promising and in line with previously reported accuracy rates for different retrosynthesis programmes.^60^ This is also consistent with results from Kerstjens and De Winter,^62^ which show similar synthesizability predictions for their LEADD generated compounds and, based on AiZynthFinder analysis of molecules in ChEMBL, suggests that AiZynthFinder may underestimate synthesizability of molecules. The neural playouts, while still promising, provided a much lower rate of predicted synthesizability. This can be explained by the fact that the neural network does not return the exact conditional probability from the training set, and thus rare moves such as the shortcuts are overrepresented in these generations. We adopted the neural network from Yang et al.^36^ with adaptations for our shortcuts and explicit bonds. While it showed worse outcomes with the implicit bonds and no shortcuts, it delivered similar results with explicit bonds and no shortcuts. However, with more fine-tuning, neural networks may have the potential to reach the same level of synthesizability as with the ngrams, although with the added disadvantage of having slower execution.

The random and enforced generations act as control experiments. As no policy governs the structure of the generation, nothing can direct the molecule generation toward a sensible and synthesizable outcome. Indeed, AiZynthFinder is unable to propose reactions, thus ending the search long before the 2 min time limit (generally in less than a second).

### Physicochemical Properties of the Generated Drug-Like Molecules

To further validate their drug-likeness, we compared the distribution of key physicochemical properties of molecules generated with NMCS with ngram playout (which generated the highest proportion of synthesizable compounds) with those generated with NMCS with random playout (used as control) and drugs retrieved from the FDA subset of ZINC-20^33^ (Supplementary File 2). For the latter, we focused on molecules abiding to the same rules used in our generation process (Lipinski’s “Rule of 5”).

**Figure 2:**
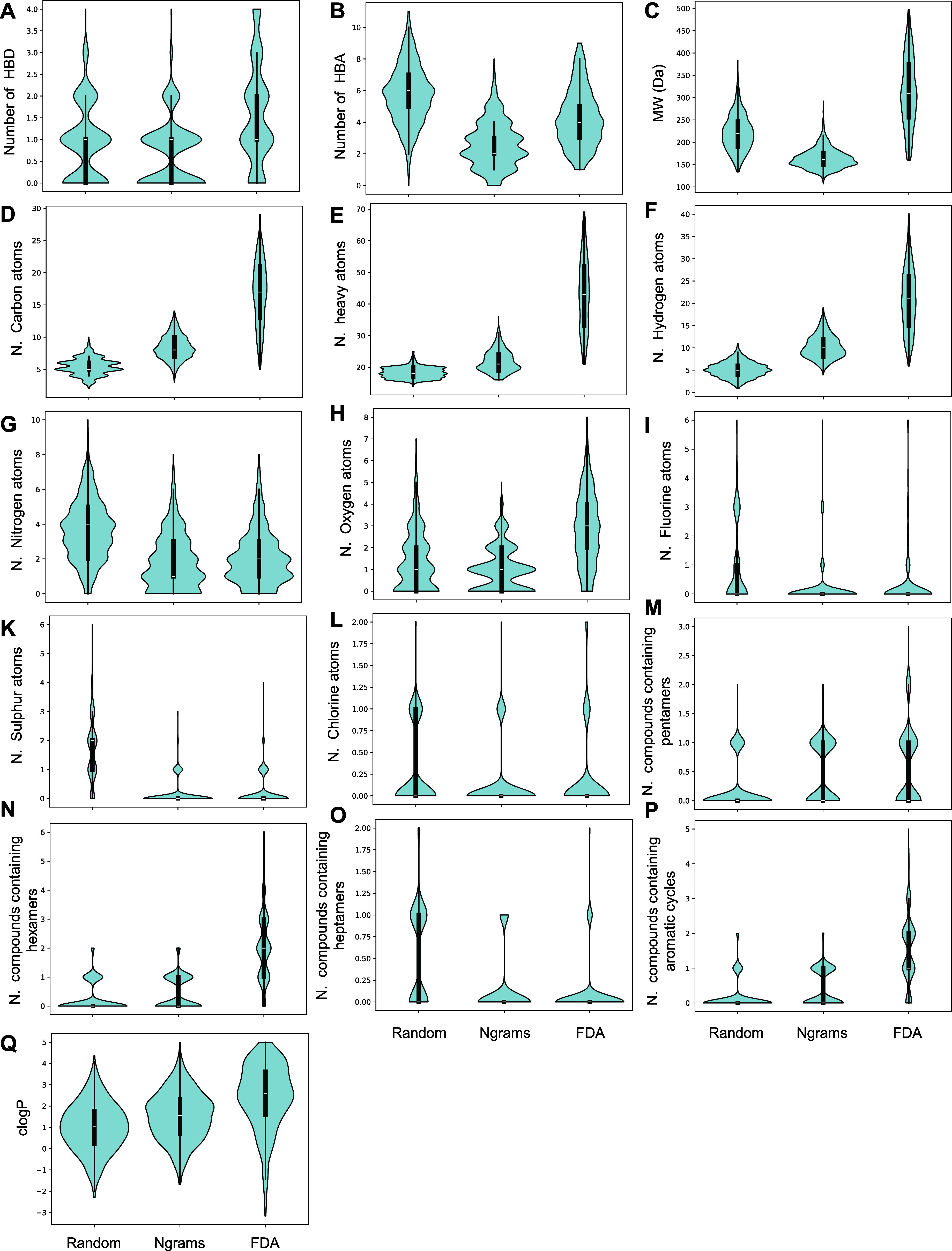
Physicochemical properties of generated drug-like Mmolecules. Comparison of physicochemical properties among drug-like compounds generated with Random and Ngram, and the “Rule of 5” drugs within the FDA subset of the ZINC20 database. Violin plots showing (A) number of hydrogen bond donors (HBD), (B) hydrogen bond acceptors (HBA), (C) molecular weight (MW), (D) total number of Carbons, (E) heavy atoms, (F) hydrogens, (G) nitrogen, (H) oxygen, (I) fluorine, (K) sulfur, (L) chlorine, (M) pentamers, (N) hexamers, (O) heptamers, (P) aromatic cycles, and (Q) clogP. Physicochemical properties were calculated on libraries containing 1,000 generated compounds.

It is been shown that, to avoid reducing oral bioavailability, the number of hydrogen bond donors (HBD) should be lower than 6 and hydrogen bond acceptors (HBA) lower than 15.^63,64^ Despite our stated upper limit of 10 for HBA and 5 for HBD, we found that compounds generated with ngram peak at 2 and 1 for HBA and HBD, respectively, with an overall lower number of HBA than random, and a higher ratio of compounds with fewer HBD than random and FDA (Figure 2A,B). By design, ngrams generate compounds with lower MW and total number of carbons (and, as a consequence, lower number of heavy atoms) and hydrogens than drugs in FDA (Figure 2C–F). This is a byproduct of the search stopping whenever the Lipinski rules are satisfied, and an indispensable requirement for (i) future optimization studies where compounds may need to be grown to adapt to pockets in targets and increase overall affinity, and (ii) synthesizability analysis with AiZynthFinder. The distribution of heavy atoms is overall comparable in all plots, as the score function generates molecules containing the same heavy atoms (nitrogen, oxygen, fluorine, sulfur, chlorine) ratios as in FDA drugs (Figure 2G–L). DrugSynthMC’s score function balances the number and size of rings (Figure 2M–O), but not their type (aromatic and aliphatic, homo- and heterocycles), based on the probability of occurrence of different rings calculated from FDA drugs. The formula returns drugs with a nearly equal distribution of compounds with zero or one aromatic cycle, which is lower than the aromatic cycle content in FDA drugs (Figure 2P). However, it has been shown that oral drugs with less than 3 aromatic rings have good compound developability,^65^ suggesting that DrugSynthMC has the potential to generate molecules with low risk of attrition in early stage development. Generated compounds also have promising oral bioavailability parameters. Earlier work by Soares et al.^66^ showed that FDA-approved drugs in the last 20 years have a relatively stable number of rotatable bonds (mean 5, median 7.5) with about 89% drugs containing less than 10 rotatable bonds. ngram playouts successfully generated compounds with less than 6 rotatable bonds (mean 3) and, in proportion, produced a higher number of molecules with fewer and higher rotatable bonds than the FDA drugs and Random playout, respectively, thus potentially identifying a balance between flexibility and diffusional cross-section (Figure 2P).

The logarithm of the octanol–water partition coefficient (logP) is a widely used parameter to define solubility of compounds in water and their upper limit of intrinsic solubility. Extensive analysis of experimental logP and calculated logP (clogP) of approved drugs over the past 30 years showed that the cutoff <5 remained constant with a mean of about 3.4.^67−69^ Unlike other rule of 5 physicochemical descriptors which are routinely calculated based on SMILES or compounds structures, the clogP is highly dependent on the calculation method, with recent approaches relying on computationally expensive and state-of-the-art neural approaches.^70^ Faster approaches are available but they trade accuracy for speed. As such, to avoid making the evaluation function computationally demanding or inaccurate, the calculation of logP was not included in the score function. Instead, the clogP was calculated on generated compounds using Rdkit, which provides an implementation of the atom-based Wildman-Crippen method.^71^ All three sets of molecules analyzed have overall similar clogP (Figure 2Q). The distribution of clogP showed that the majority of molecules have a value between −2 and 4, with ngram showing a larger number of compounds with lower clogP (∼2) than that of the FDA, suggesting high likelihood of these compounds being orally bioavailable. Water solubility can also be conveniently estimated using SwissADME which relies on the ESOL model^72^ to classify SMILES. Consistent with clogP analysis, all SMILES are classified as soluble, very soluble, and highly soluble (Supplementary File 3).

### Pharmacokinetics and ADMET Predictions (Absorption, Distribution, Metabolism, Excretion, and Toxicity) of the Generated Drug-Like Molecules

Pharmacokinetics and ADMET properties of the generated compounds were assessed by predicting metrics commonly employed in drug discovery: (i) the quantitative estimate of druglikeness (QED) score^73^ and similarity with FDA drugs; (ii) the synthetic accessibility (SA) score;^32^ (iii) proportion of pan-assay interference compounds (PAINS) (i.e., structural alerts likely to produce false positives in *in vitro* assays);^74^ (iv) predicted metabolism and toxicity, defined as the likelihood to inhibit one of the five isoforms of cytochromes P450^40^ and an extensive subset of toxicity end points.^41^

As a result of the function of compliance adopted to design DrugSynthMC, ngrams-generated compounds are characterized by a high druglikeness (average QED 0.53 ± 0.11) and low violation (Supplementary File 3) of the five rule-based filters implemented in SwissADME.^40^ Importantly, while being drug-like, they are novel. In fact, the estimated similarity between ngrams-generated compounds and FDA drugs, estimated using the Tanimoto index^75^ computed between each generated molecule and the FDA drugs, is very low with most compounds having a Tanimoto index lower than 0.3 (Supplementary Figure 1).

To expand synthesizability analysis, we estimated the SAscore, which relies on historical synthetic knowledge obtained by analyzing synthesized chemicals and adds penalty for molecular complexity, with values ranging from 1–5 (easy to synthetize) to 6–10 (difficult to synthetize).^32^ In line with results obtained for the retrosynthesis analysis, SAscore shows that all generated drug-like molecules are predicted to be easily synthesizable, with no significant differences with the FDA subset (Supplementary Figure 2). As expected, the ngram-generated compounds perform better in terms of SAscore than those produced by the random method. It is worth noting that the SAscores obtained for the ngram-generated compounds are perfectly comparable with published SAscores from other algorithms. For example, Popova et al.^52^ showed that the median SAscore for one million compounds generated with ReLeaSE is of 3.1, while the mean SAscores for 700,000 DeLA-Drug generated molecules or 1000 AlphaDrug-generated drugs is 2.9.^51,76^ Consistently, DrugSynthMC synthesizability analysis is also in line with SBMolGen, which filters out during the design stage any molecules with SAscores greater than 3.5.^28^

DrugSynthMC generates also a very low percentage (ngrams 4.7%) of molecules predicted to be PAINS (Supplementary File 3), comparable with results from other approaches.^51^ Similarly, ngrams-generated SMILES tend to be molecules that can be metabolized by most isoforms of cytochromes P450, with no compounds inhibiting all five tested isoforms and only 4% inhibiting 2 or more isoforms (Supplementary File 3). Furthermore, only 0.03% compounds are predicted to be substrate of the permeability glycoprotein, which plays a key role in active efflux of compounds outside of the cell, driving drug resistance in some types of cancers.^77^ The toxicity profile varies according to the subset of toxicity end point considered, but in general Deep-PK analysis of ngram-generated SMILES is comparable to that of the FDA (Supplementary File 4), with about 80% compounds predicted to be safe for the liver.

This supports the robustness of the design principle and the suitability of generated compounds for real drug design applications.

## Conclusions

DrugSynthMC is capable of designing novel and chemically diverse drug-like compounds by generating character-by-character SMILES. We compared different algorithms with different payouts. Importantly, we showed that the ngram playout is superior to the more advanced neural approaches. Very likely, this is linked to the ability of ngrams to follow more closely the distribution of atoms of preexisting valid molecules. Furthermore, as expected, NMCS outperforms UCT in random and enforced generation, as NMCS has usually previously been found to perform better than UCT on optimization problems.^78,79^ Crucially, DrugSynthMC does not rely on training data sets, thus generating drug-like compounds in a robust, and reliable way, independently from both the complexity and diversity of targets. DrugSynthMC is fast and highly flexible and in the future could be easily tuned to generate customized drug libraries tailored for specific binding pockets on targets. For example, DrugSynthMC libraries could be tested using our previously adopted ensemble screening approach^80^ and generated pseudofree energy of binding could be fed as a reward in a reinforcement learning method within DrugSynthMC.^81^ Convergence to a minimal value of pseudofree energy of binding over several iterations of DrugSynthMC is likely to highlight a set of compounds with the desired pharmacophores and binding affinities.

## Data Availability

Our algorithm, DrugSynthMC, is freely accessible on Github at https://github.com/RoucairolMilo/DrugSynthMC. The exemplar SMILES generated by DrugSynthMC and used for all analysis presented in this manuscript are provided as Supplementary File 1.

## Supporting Information Available

Exemplar SMILES generated by DrugSynthMC and used for all analysis presented in this article (PDF)
Distribution of key physicochemical properties of molecules generated with NMCS with ngram playout (which generated the highest proportion of synthesizable compounds) with those generated with NMCS with random playout (used as control) and drugs retrieved from the FDA subset of ZINC-20 (XLSX)
Classification of SMILES (XLSX)
ngrams-generated compounds characterized by a high drug-likeness (average QED 0.53 ± 0.11) and low violation of the five rule-based filters implemented in SwissADME (XLSX)
DrugSynthMC generates also a very low percentage (ngrams 4.7%) of molecules predicted to be PAINS, comparable with results from other approaches (XLSX)
Table S1: Time (s) to generate 1000 molecules (PDF)
Table S2: Synthesizability of 1000 NMCS-generated SMILES with different playouts computed by AiZynthFinder in 2 minutes (PDF)
