# Supplementary material for: DrugSynthMC: An Atom-Based Generation of Drug-like Molecules with Monte Carlo Search
Source: J Chem Inf Model. 2024 Sep 9;64(18):7097–107. doi: 10.1021/acs.jcim.4c01451 (PMC11423341; doi:10.1021/acs.jcim.4c01451)
Supplement: Supplementary file 1 — ci4c01451_si_001.pdf [file ci4c01451_si_001.pdf]

DrugSynthMC: an atom-based generation of drug-like molecules with Monte Carlo Search

Milo Roucairol[1], Alexios Georgiou[1], Tristan Cazenave[1*], Filippo Prischi[2*], Olivier E. Pardo[3*].

[1] LAMSADE, Université Paris-Dauphine, Pl. du Maréchal de Lattre de Tassigny, 75016 Paris, France

[2] Randall Centre for Cell and Molecular Biophysics, School of Basic and Medical Biosciences, King's College London, London SE1 1UL, United Kingdom.

[3] Division of Cancer, Department of Surgery and Cancer, Imperial College, Du Cane Road, London W12 0NN, United Kingdom

* Corresponding authors:
Tristan Cazenave - tristan.cazenave@lamsade.dauphine.fr
Filippo Prischi - filippo.prischi@kcl.ac.uk
Olivier E. Pardo – o.pardo@imperial.ac.uk

Supplementary Figure 1

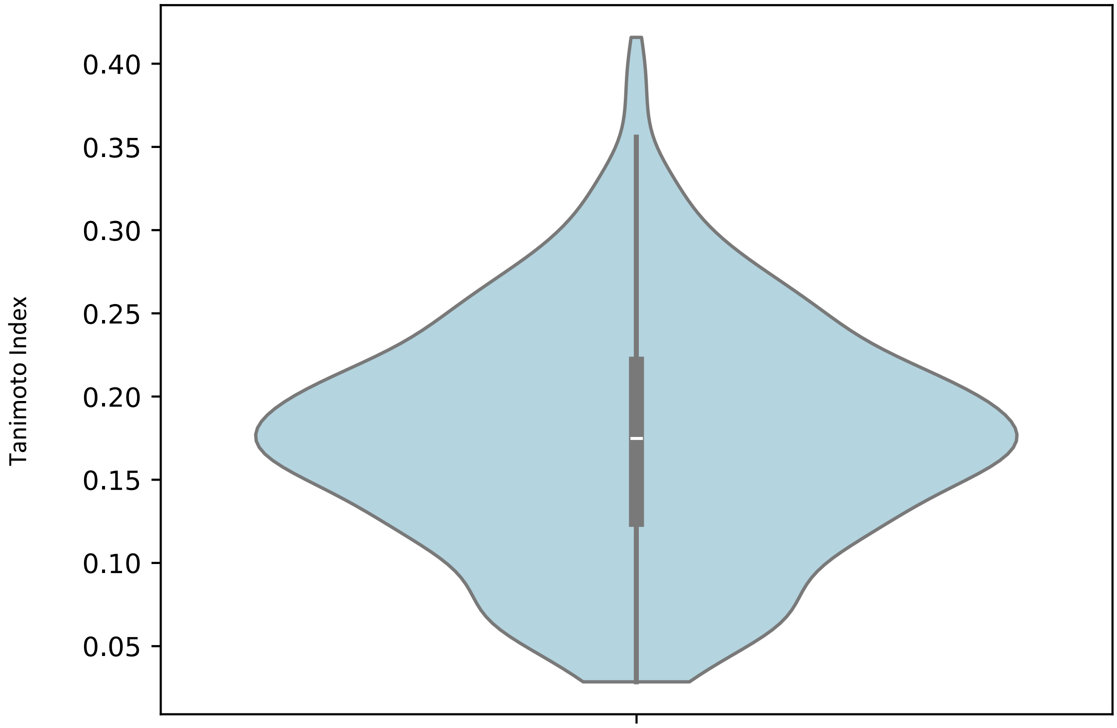

The similarity between 10,000 ngrams-generated compounds and the FDA drugs was estimated using the Tanimoto index calculated in RDKit.

Supplementary Figure 2

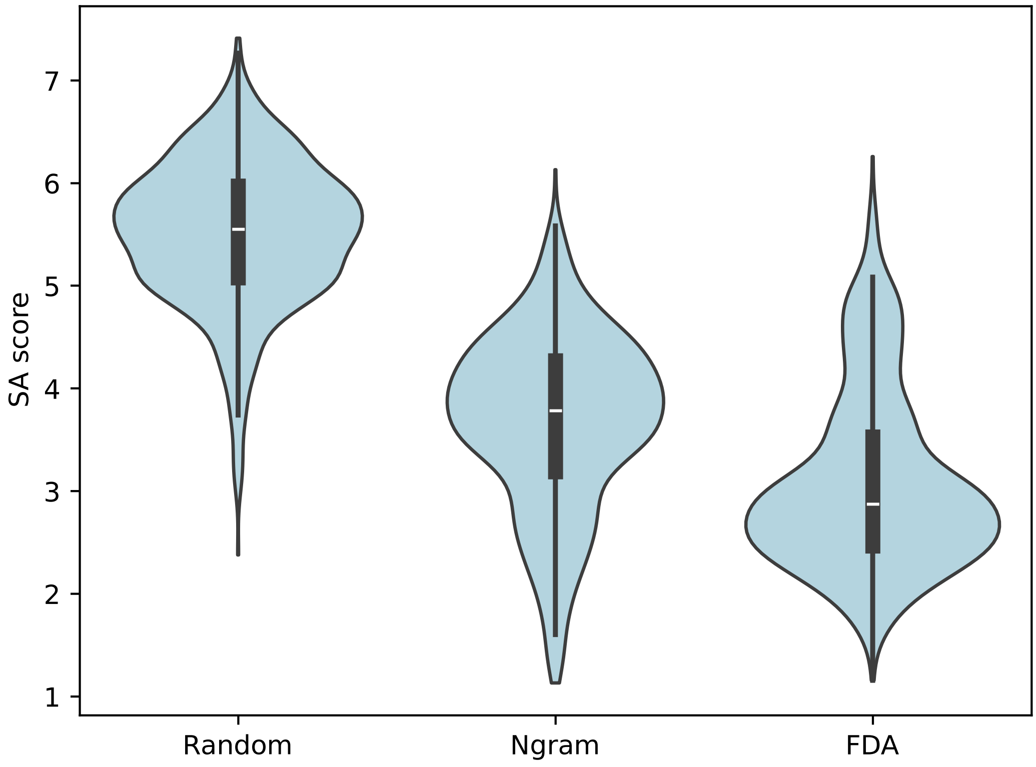

The Synthetic Accessibility score (SAscore) for 10,000 Random-generated compounds, 10,000 ngram-generated compounds and FDA drugs was calculated using RDKit.
