# Supplementary material for: DrugSynthMC: An Atom-Based Generation of Drug-like Molecules with Monte Carlo Search
Source: J Chem Inf Model. 2024 Sep 9;64(18):7097–107. doi: 10.1021/acs.jcim.4c01451 (PMC11423341; doi:10.1021/acs.jcim.4c01451)
Supplement: Supplementary file 7 — ci4c01451_si_007.pdf [file ci4c01451_si_007.pdf]

DrugSynthMC: an atom-based generation of drug-like molecules with Monte Carlo Search

Milo Roucairol[1], Alexios Georgiou[1], Tristan Cazenave[1*], Filippo Prischi[2*], Olivier E. Pardo[3*].

[1] LAMSADE, Université Paris-Dauphine, Pl. du Maréchal de Lattre de Tassigny, 75016 Paris, France

[2] Randall Centre for Cell and Molecular Biophysics, School of Basic and Medical Biosciences, King's College London, London SE1 1UL, United Kingdom.

[3] Division of Cancer, Department of Surgery and Cancer, Imperial College, Du Cane Road, London W12 0NN, United Kingdom

* Corresponding authors:
Tristan Cazenave - tristan.cazenave@lamsade.dauphine.fr
Filippo Prischi - filippo.prischi@kcl.ac.uk
Olivier E. Pardo – o.pardo@imperial.ac.uk

Table S2: Synthesizability of 1000 NMCS-generated SMILES with different playouts computed by AiZynthFinder in 2 minutes.

	random	enforced	ngram	neural
NMCS	0.4%	0.9%	32.2%	18.1%
